# Synthesis of Composite Sorbents with Chitosan and Varied Silica Phases for the Adsorption of Anionic Dyes

**DOI:** 10.3390/molecules29092087

**Published:** 2024-05-01

**Authors:** Magdalena Blachnio, Malgorzata Zienkiewicz-Strzalka, Anna Derylo-Marczewska

**Affiliations:** Faculty of Chemistry, Maria Curie-Sklodowska University, M. Curie-Sklodowska Sq. 3, 20-031 Lublin, Poland; magdalena.blachnio@mail.umcs.pl (M.B.); malgorzata.zienkiewicz-strzalka@mail.umcs.pl (M.Z.-S.)

**Keywords:** chitosan–silica composite, acid red 88 adsorption, acid orange 8 adsorption, orange G adsorption, adsorption kinetics

## Abstract

In this work, various types of silica materials were used for the synthesis of chitosan–silica composites. The composites were obtained using the chitosan (Ch) immobilization process from an aqueous solution on various silica phases, i.e., amorphous diatomite (ChAD), crystalline diatomite (ChCD), mesoporous silica MCM-41 (ChMCM), and mesoporous silica SBA-15 (ChSBA). Textural, structural, morphological, and surface properties of the materials were determined by using various measurement techniques, i.e., low-temperature adsorption/desorption isotherms of nitrogen, X-ray diffraction (XRD), small-angle X-ray scattering (SAXS), potentiometric titration, high-resolution transmission electron microscopy (HRTEM), scanning electron microscopy (SEM), and atomic force microscopy (AFM). The adsorption properties towards various anionic dyes, i.e., acid red 88 (AR88), acid orange 8 (AO8), and orange G (OG), were evaluated based on kinetic and equilibrium measurements. The ChSBA, ChAD, and ChMCM composites were characterized by relatively high adsorption capacities (a_m_) for AR88, with values equal to 0.78, 0.71, and 0.69 mmol/g, respectively. These composites were also distinguished by the rapid AR88 adsorption rate, with the values of half-time parameter t_0.5_ equal to 0.35, 2.84, and 1.53 min, respectively. The adsorption equilibrium and kinetic data were analyzed by applying the generalized Langmuir isotherm and the multi-exponential equation (m-exp), respectively. An interaction mechanism between the dyes and the obtained materials was proposed.

## 1. Introduction

The dyeing industry is a significant contributor to environmental pollution due to the discharge of wastewater containing various chemicals into the water environment [1,2,3]. The dyeing process involves the use of large quantities of water and various chemicals, including dyes, mordants, and auxiliary chemicals. Thus, after dyeing, wastewater containing residual dyes and various types of other chemicals is generated. The problem with using dyes is that they can have adverse effects on aquatic ecosystems and organisms [4,5,6,7,8,9,10]. They may reduce light penetration into water bodies, thereby inhibiting photosynthesis and disrupting the aquatic food chain. Dyes can also be toxic to aquatic organisms, affecting their growth, reproduction, and survival. Moreover, wastewater may contain various chemical additives used in the dyeing process, such as surfactants, fixing agents, and pH adjusters. Such chemicals can further contribute to environmental pollution and may have harmful effects on aquatic organisms and ecosystems [11,12,13].

Due to growing environmental problems and the development of the textile industry, various treatment methods can be used to remove dyes from water, including physical, chemical, and biological processes [14,15,16]. Common techniques include coagulation and flocculation [17,18,19,20,21,22,23], membrane filtration [24,25,26,27,28,29], biological degradation [20,30,31,32,33,34], advanced oxidation processes, and adsorption [35,36]. The adsorption process is widely used due to its effectiveness and relatively simple implementation [37,38,39,40]. The choice of treatment method depends on factors such as the type of dye, its concentration, and the desired level of treatment. In recent years, there has been growing interest in the development of advanced treatment technologies for removing dyes from water. These may include photocatalysis [41,42,43,44,45], electrochemical oxidation [46,47,48,49], and nanomaterial-based approaches [50,51,52,53], which offer potential advantages such as high efficiency, low energy consumption, and the ability to degrade a wide range of dye compounds. Nanomaterials can be modified by adding functional chemical groups that increase their ability to adsorb dyes, chemicals, and contaminants through chemical interactions [54,55,56,57,58,59]. Nanomaterials based on chitosan represent a versatile platform with a wide range of potential applications [13,60,61,62,63]. Ongoing research continues to explore new synthesis strategies and functionalizations to further enhance the performance and applicability of these composites. Over the last two decades, the demand for natural bioactive materials has increased as they are non-toxic and safer than synthetic polymers [64,65,66]. The search for new biodegradable polymers, alternative sources, and renewable fillers that enable the production of environmentally friendly and functional biocomposites is still ongoing. Chitosan has a very wide range of applications. Cases of its use in adsorption applications include the following works [67,68,69,70]. A major advantage of biopolymers, including chitosan, is the ability to combine with many anionic polymers and the possibility of molding them into various functional forms with significant porosity in the structure (including hydrogels, sponges, fibers, and nanofibers) [71,72,73,74,75]. The applicability of a biopolymeric material without the addition of modifiers is often problematic, so biopolymer systems with other types of materials with specific nanostructural and physicochemical properties are being considered. Among the whole range of possibilities, nanostructured silica phases and naturally occurring porous sedimentary rock (diatomite) seem to be interesting types of modifiers [76,77,78]. Nanometric silica is a promising composite filler that should be considered in the development of new types of functional materials. This is because it combines the most important properties of both inorganic materials and systems with unique nanostructure and porosity. In addition, it is readily available with a variety of structural modifications. When silica phases are incorporated into chitosan matrices, they can improve mechanical strength and thermal stability and provide antimicrobial properties [79,80,81]. These composites find applications in drug delivery, tissue engineering, and wound healing [82,83,84,85,86,87]. Mesoporous silica–chitosan composites are of particular interest due to their unique combination of properties, including high surface area, tunable pore size, biocompatibility, and mechanical strength. Some advantages of mesoporous silica–chitosan composites include the ability to tune the pore size of the material by selecting appropriate mesoporous silica templates. This tunability allows for the control of drug release kinetics and the adsorption capacity of the composite for different applications [88,89,90]. The mesoporous structure provides a large surface area for drug loading, while chitosan offers biocompatibility and controlled release properties. The incorporation of diatomite into chitosan matrices may further enhance the antimicrobial properties and final biocompatibility. In both cases, the inherent antimicrobial properties can be beneficial for applications where microbial growth needs to be controlled (water treatment and water purification). The biopolymer–silica composite materials were evaluated on a laboratory scale for use in the adsorption process for the purification of water polluted with dyes. Dyes belonging to sulfonic azo compounds of an anionic nature, i.e., acid red 88 (AR88), acid orange 8 (AO8), and orange G (OG), can be selected as exemplary coloring pollutants [91,92,93]. These substances are used in many industries (textiles, pulp and paper, chemicals, food, and cosmetics) and are therefore an important component of industrial effluents that pose a threat to the natural environment [94,95]. On the other hand, dye molecules are highly resistant to biological, physical, and chemical degradation due to their complex structure. Therefore, the development of adsorbents that would be effective in the purification of water from these types of pollutants using adsorption technology is highly desirable. The composites that are the subject of the research contain two components: environmentally neutral silica and chitosan. The biopolymer deserves special attention due to the high availability of the material from which it is made (chitin), its biodegradability, and the unique adsorption properties associated with the polycationic nature of its macromolecules. This last property of chitosan leads to it being treated as a superior component of composites and playing a key role in the extraction of anionic dyes. Although silica is quantitatively the dominant component of the composite, it does not participate in adsorption but merely serves as a carrier for the organic phase. Silica also gives the composite better mechanical resistance and thermal stability than pure chitosan.

In the paper, two groups of silica materials were used: synthetic and natural silica materials characterized by a high proportion of mesopores (SBA-15 and MCM-41) and low-porosity materials (amorphous and crystalline diatomaceous earth). The selected materials differ in their morphology, and the results of low-temperature nitrogen adsorption indicate significant differences even within the pore structure. The use of different types of silica materials makes it possible to obtain more complementary information on the influence of the nature of the silica phase on the adsorption process of the dyes, and this was carried out for all systems with the same type of adsorbate and under the same conditions. A similar complementary approach of using various systems of a similar nature has been used in literature reports [96,97]. Various techniques, including X-ray diffraction, small-angle X-ray scattering, high-resolution transmission electron microscopy, scanning electron microscopy, atomic force microscopy, low-temperature adsorption/desorption isotherms of nitrogen, and potentiometric titration, were applied to determine the structural, morphological, textural, and surface properties of the obtained materials. The adsorption effectiveness of the composites towards selected anionic dyes was studied based on equilibrium and kinetic data. The experimental isotherms and concentration profiles were analyzed by using some chosen equations and models.

## 2. Results and Discussions

### 2.1. Material Characterization

**Figure 1:**
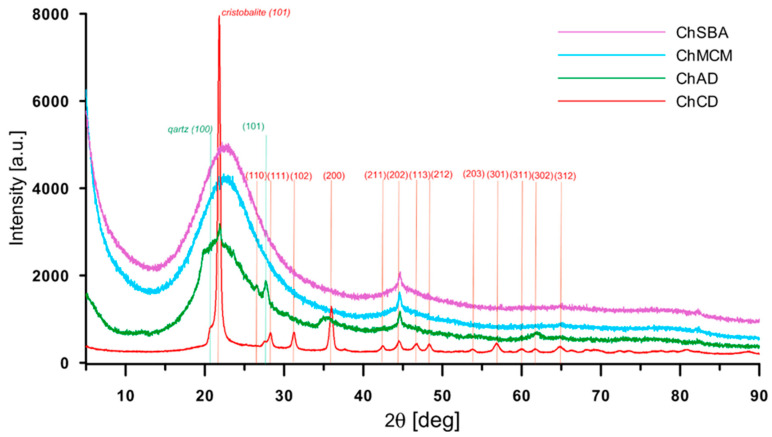
Powder XRD pattern of chitosan–silica composites (ChSBA, ChMCM, ChAD, and ChCD) with the identification of cristobalite and quartz phases in diatomite-based composites.

The X-ray powder diffraction (XRPD) technique was applied for the identification of both crystalline and amorphous phases and the determination of specimen purity. The XRPD pattern of the diatomite-based composite (ChCD) was consistent with the crystalline silica phases of cristobalite (JCPDS PDF No. 39-1425) and quartz (JCPDS PDF No. 46-1045) (Figure 1). In the composite material containing crystalline diatomite, the cristobalite silica was formed during the heating process. In the case of the ChAD composite material, the amorphous form of silica was observed. Amorphous silica (SiO_2_) was the dominant form; however, diffraction peaks of the crystalline phases were still visible. For ChSBA and ChMCM samples, the broad peak at 15–30° was ascribed to amorphous silica and was typical for mesoporous silica phases [98,99].

**Figure 2:**
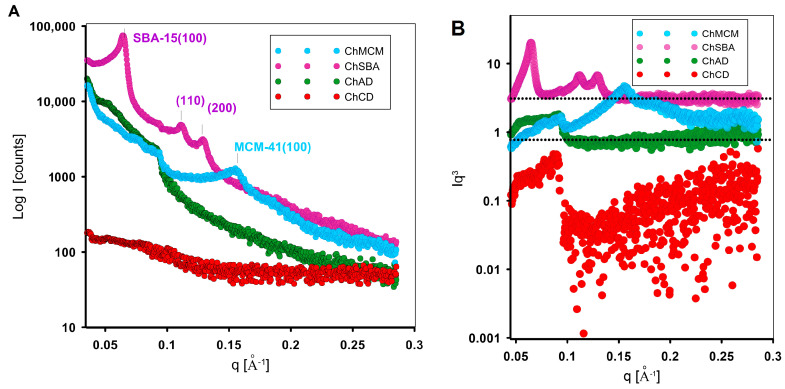
SAXS pattern of chitosan–silica composites (ChSBA, ChMCM, ChAD, and ChCD): (**A**) Porod plots for investigated samples; (**B**) Porod curves are presented on a logarithmic scale for better visibility.

The structural ordering of the ChSBA and ChMCM composites, as well as the structural analysis of all investigated samples, was analyzed by small-angle X-ray scattering. For the SAXS patterns, Figure 2A shows the relationship between the scattering intensity (I) and the scattering vector (q) and confirms that mesoporous materials (ChSBA and ChMCM) exhibit structural order. For the ChSBA sample, three well-resolved peaks related to the (100), (110), and (200) planes were confirmed to be associated with long-range 2D hexagonal ordering in the p6mm space group. These results are consistent with the data reported by Zhao [100]. The quality of the ordered structure in the case of the ChSBA biopolymer–silica material was satisfactory. The positions of peaks (100), (110), and (200) were determined to be q = 0.065 Å^−1^, 0.1124 Å^−1^, and 0.130 Å^−1^. The unit cell parameters calculated from the position of the (100), (110), and (200) peaks corresponded to 111.73 Å, 111.74 Å, and 111.56 Å, respectively (*a*_0avg_ = 111.7 Å). The comparison of the SAXS spectra of ChSBA and ChMCM composite materials with SAXS curves of initial SBA-15 and MCM-41 is presented in Figure S1.

The well-defined signals on the SAXS curve confirm that the introduction of the biopolymer component allows the maintenance of an ordered structure characteristic of the SBA-15 type silica phase. In the case of the ChMCM material, the structural order was somewhat weaker and was reflected by the presence of only one characteristic signal (100). In this case, the hexagonal phase was associated with the shorter repeat distance *a*_0_ = 48.3 Å. The presence of a biopolymer phase in the system of small pores of the silicate support can be attributed to a degradation in the mesoscopic pore order to a greater extent than in the case of the ChSBA material containing larger pores. In the biopolymer–diatomite composites, differences in the intensity of the X-rays were observed as a function of the degree of crystallinity of the diatomite phase. While the composite material obtained from diatomite in amorphous form showed a certain scattering intensity, the crystalline form had almost no such effect. In both cases, the SAXS curve did not indicate the presence of ordered phases.

An attempt was made to analyze the quality of the chitosan layer on a silica support. The Porod curves (Figure 2B) show a positive deviation for the ChCD sample. This positive deviation is due to the nonuniform area of electron density in the chitosan–diatomite sample according to the nonideal two-phase system. Here, density fluctuations in the phases (interphase area) generate a deviation of Porod’s law, evidenced by a positive slope of the linear range (high q-values). In other cases, the interface showed no significant blurring elements. Only for the ChMCM sample was there evidence of a slight negative deviation from the linearity law, which could be related to a specific transition region between the biopolymer and the silica phase. However, the influence of the presence of residues of the silicon dioxide material matrix cannot be ruled out, especially as this material exhibits small pore sizes.

**Figure 3:**
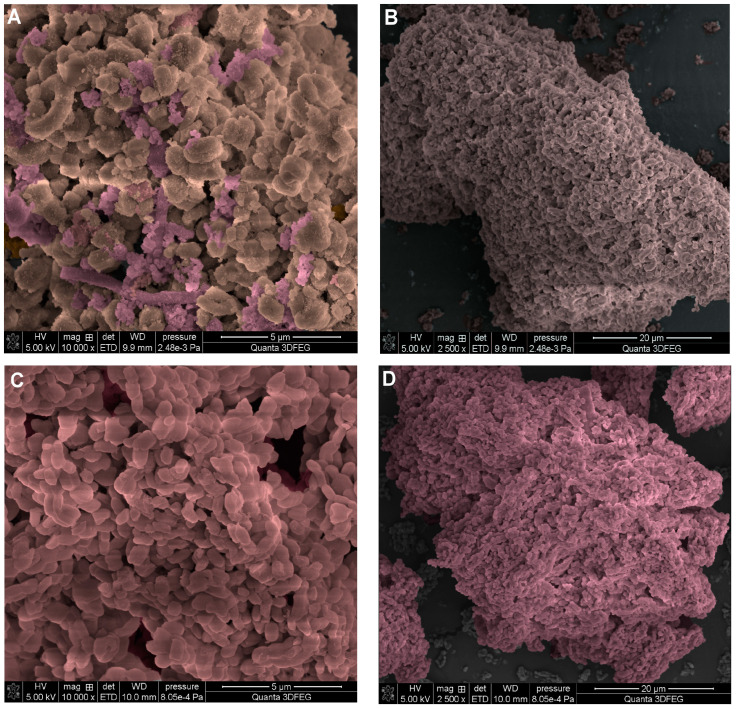

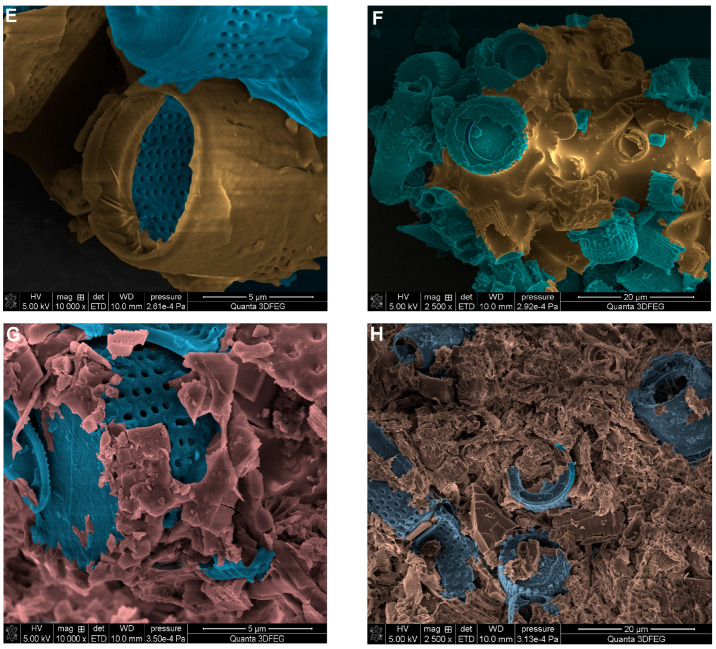
SEM images of chitosan–silica composites: ChMCM (**A**,**B**), ChSBA (**C**,**D**), ChCD (**E**,**F**), and (**G**,**H**) ChAD. Each sample is presented at two magnifications (10.000× and 2500×). The original SEM images without colorization are included in the SM file (Figure S2).

Microscopic analysis has been used in the study of silica–silica composites as a key tool in materials research to understand their morphology, properties, the quality of the composite form, and the distribution of components, as well as possible defects. The microscopic analysis aimed to investigate the presence and possibly the shape of the chitosan material in a system with silica materials of different types. By analyzing SEM images, the presence of a distinct surface chitosan phase on the surface of the ChMCM sample can be confirmed. Chitosan can form small fragments with a rough surface texture that appear as agglomerates of different sizes and shapes, as well as microfibrils (marked in pink object in Figure 3A,B). ChSBA materials show a short rod-shaped morphology with uniform sizes (from 300 nm to 500 nm). The SEM images of the ChSBA sample (Figure 3C,D) show that the surface of the silica particles is more homogeneous than that of ChMCM. The chitosan phase was not identified in the form of agglomerates or precipitates.

Figure 3E,F show an SEM image of the ChCD sample. The photographs present a mineral composed of siliceous fragments of various types of fossilized diatom remains. SEM photos prove the biogenic origin of the material, which is confirmed by skeletal forms of diatoms in the form of rods, flakes, and cylinders. In addition, open and pollution-free pores with a size of 250–500 nm are visible in the shell. In this case, a thin film-like layer was observed (Figure 3F) which may be associated with the biopolymer component. Such a surface covering of siliceous forms was visible in several photographs of this sample (only two were selected for presentation in this paper). The sample ChAD (Figure 3G,H) is a system of highly fragmented shells (skeletons) of diatoms. For this sample, it was not possible to identify the area associated with chitosan from SEM images.

**Figure 4:**
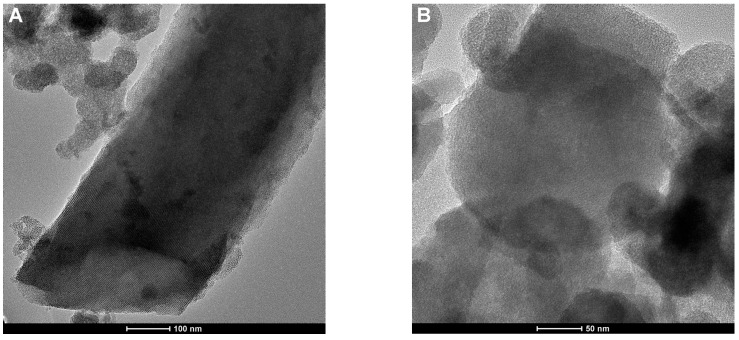

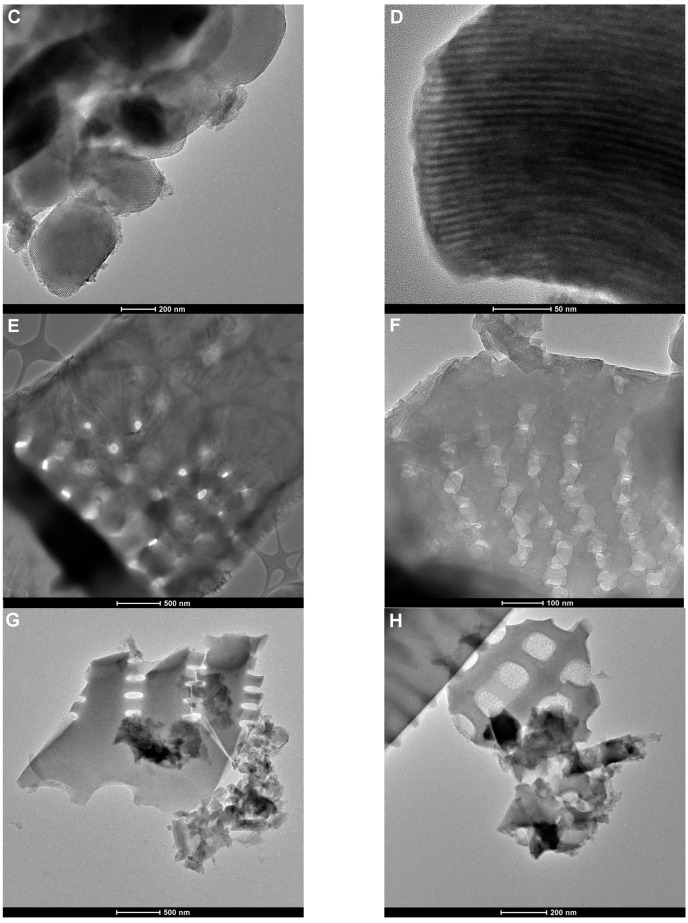
TEM images of chitosan–silica composites: ChMCM (**A**,**B**), ChSBA (**C**,**D**), ChCD (**E**,**F**), and (**G**,**H**) ChAD.

The TEM images exhibited an array of well-ordered mesopore channels and a well-defined porosity of both ChMCM and ChSBA composites (Figure 4A–D). The chitosan–silica materials ChMCM and ChSBA are characterized by the presence of regular pores, usually between 2 and 50 nanometers in diameter. TEM images confirmed their shape and regularity. In addition to these characteristic features of the silica phase, there are also areas on TEM images that do not show such regularity. In addition, the location of the mesoporous silica particles on the surface may indicate that it is a phase of a different nature, forming a thin film on the silica surface. It is to be expected that this is the chitosan phase, which does not completely cover the surface of the silica material. The TEM observation of chitosan–diatomite (Figure 4E–H) is a complement to the SEM results in evaluating the general morphology and the presence of the pores, as well as in evaluating their shape and size. In addition, these photos show a thin layer of amorphous phase partially covering the pores of the starting material. The thin film on the surface of the material could be a biopolymer layer.

**Figure 5:**
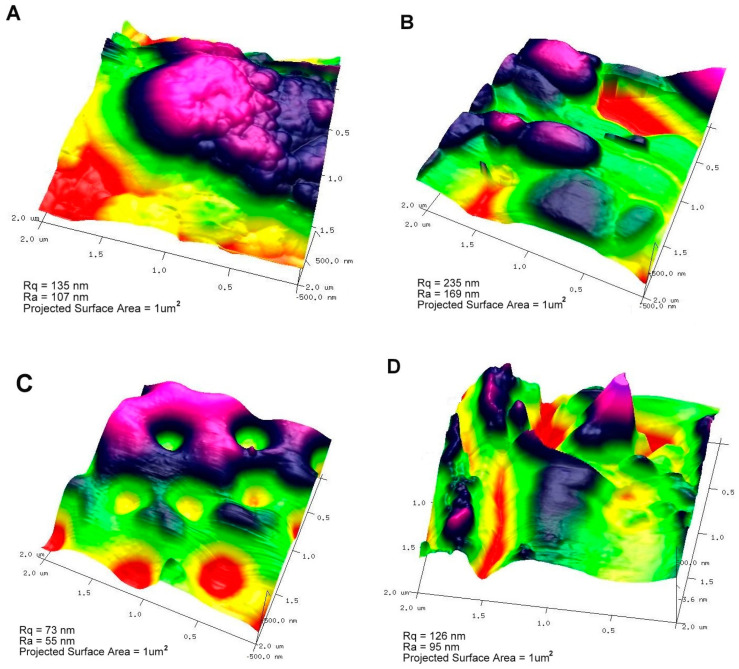
Atomic force microscopy (AFM) topography images of chitosan–silica composites: ChMCM (**A**), ChSBA (**B**), ChCD (**C**), and ChAD (**D**).

The amorphous layer of chitosan on the silica phase was analyzed using atomic force microscopy. The surface roughness of the selected area (1 μm × 1 μm of the flattest area of the AFM image) is presented under each topography. In the case of the ChMCM sample, the surface was rough (on the surface of the silica grain: Figure 5A). The surface of the ChSBA sample was rougher (Ra = 169 nm and Rq = 235); however, this is influenced by the nature of the silica grains themselves (Figure 5B). The surface roughness of the grain itself (300 nm × 300 nm) is similar to that of ChMCM and equal to R_a_ = 166 nm and R_q_ = 229. This may reflect the similar nature of the chitosan covering layer, which is present to a lesser extent in the diatomite phase (Figure 5C,D).

**Figure 6:**
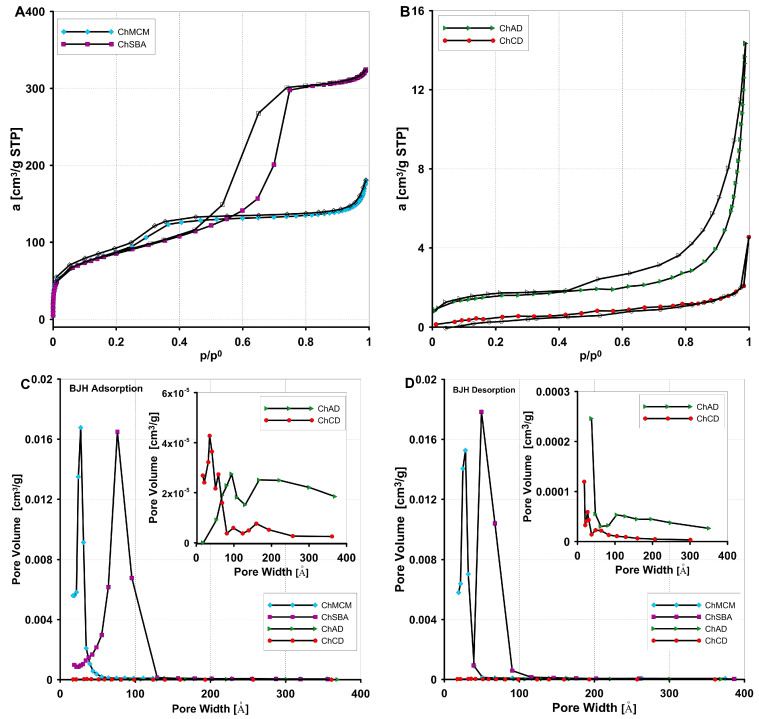
Nitrogen adsorption/desorption isotherms for the ChMCM, ChSBA composites (**A**) and the ChAD, ChCD composites (**B**). Comparison of pore volume distributions vs. their sizes for composites determined from the adsorption (**C**) and desorption (**D**) branches of gas isotherms.

To perform a textural analysis of the obtained chitosan–silica composites, nitrogen adsorption/desorption isotherms at a temperature of 77 K were measured (Figure 6A,B). The pore volume distribution functions (PSD) were determined using the method developed by Barrett, Joyner, and Halenda (BJH) (Figure 6C,D). Taking into account the textural properties of the synthesized materials, they can be classified into two groups, namely porous ones (ChSBA, ChMCM) and those characterized by low porosity (ChAD, ChCD). According to the IUPAC classification, the adsorption isotherm for ChSBA is type IV with a type H1 hysteresis loop, which is characteristic of mesoporous materials with cylindrical pores. The shape of the isotherm for ChMCM is similar to the type IV isotherm, with a hysteresis loop shifted towards lower relative pressures. The PSD analysis shows that the pore distribution functions represent a homogeneous (Gaussian) type with a main peak in the region of the smaller mesopores, with diameters of about 8 nm and 3 nm for ChSBA and ChMCM, respectively. Both composites are characterized by a comparable specific surface area (303 and 330 m^2^/g) (Table 1), although the values of their total pore volume are significantly different (0.49 and 0.26 cm^3^/g). The twice-as-high V_t_ parameter for ChSBA results from the larger proportion of pores with larger diameters compared to ChMCM. In the second group of materials, the gas adsorption/desorption isotherm for ChAD corresponds to mixed type II and IV with a hysteresis loop of type H3 (slit pore shape), while the isotherm for ChCD represents type II. The course of the isotherms with a significant increase in gas adsorption at relatively high relative pressures (p/p^0^ > 0.8) indicates that these materials have small amounts of pores of higher size in the mesopore/macropore region. This is confirmed by the PSD distributions, which are heterogeneous with several pore radius maxima, and broader in the region of the larger pore diameters. The values of specific surface area (5.2 and 2.1 m^2^/g for ChAD and ChCD, respectively) and total pore volume (0.02 and 0.01 cm^3^/g for ChAD and ChCD, respectively) are significantly lower than those for the other composites. Such a low porosity of the ChAD and ChCD composites is a consequence of the use of naturally occurring silicas (in uncalcined and calcined forms), characterized by only slightly better textural parameters. According to Marczyk and co-workers [101], the specific surface area of unmodified diatomaceous earth is about 27 m^2^/g, and it decreases after the material has been subjected to the calcination process. It was observed that the higher the calcination temperature, the lower the specific surface area of the final product, i.e., S_BET_ equals 22 m^2^/g and 2 m^2^/g for calcined diatomite samples at 650 °C and 1000 °C, respectively.

The obtained composite materials were analyzed to determine the content of selected elements (carbon, hydrogen, and nitrogen). Two measurements were performed for each sample and the results shown in Table 2 are the average values. Despite the use of a single synthesis method for all composites and a constant weight ratio of chitosan to silica (1:5), some differences in elemental composition were observed. The nitrogen content derived from the nitrogen functional groups (amine and amide) allowed the quantitative determination of the biopolymer content in the adsorbents. For the three samples (ChSBA, ChMCM, and ChAD), the nitrogen content was similar or equal to 1–1.3%, while for the ChCD sample, it was about half as high (0.6%). This disproportion may result from the incomplete binding of the biopolymer used with crystalline diatomite. The latter is characterized by a low specific surface area, which results in a limited contact surface of the two phases, i.e., the chitosan solution and solid, and ultimately leads to chitosan adsorption in a smaller amount. Unbound chitosan was removed during the decantation stage.

It can be concluded that the actual composition of the final products is different. The highest proportion of chitosan component was found in ChSBA and ChAD, with slightly lower results in ChMCM, and the lowest in ChCD.

**Figure 7:**
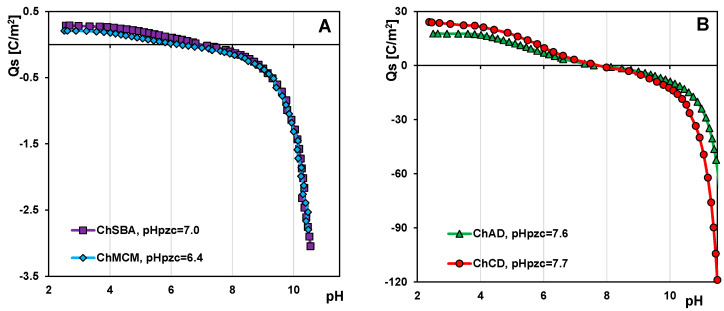
Dependences of surface charge density on pH for the ChMCM, ChSBA composites (**A**) and the ChAD, ChCD composites (**B**).

To characterize the acid–base properties of the surface of chitosan–silica composites, potentiometric titration measurements were performed. The data obtained were used to determine the values of the point of zero charge (PZC) of the adsorbents and the changes in surface charge density as a function of solution pH (Figure 7A,B). The point of zero charge refers to the state of a solid particle in which the sum of positive and negative charges accumulated on its surface is zero and the pH of the solution responsible for this state is pH_pzc_. When the pH of the suspension of solid particles is below PZC, conditions favoring the protonation of surface groups occur, resulting in a positive charge on the solid surface. Above PZC, the opposite trend is observed and the solid surface acquires a net negative charge. In the case of obtained materials, the pH_pzc_ value depends on the following factors: (i) the relative proportion of the components; (ii) the acidity of the silica used; and (iii) the mineral composition if diatomite was used for the synthesis. The values of the parameter discussed were 6.4, 7.0, 7.6, and 7.7 for ChMCM, ChSBA, ChAD, and ChCD, respectively. The pH_pzc_ values for ChMCM and ChSBA correlate well with the nitrogen content in these samples. The higher the nitrogen content (due to the presence of the polycationic chitosan component), the higher pH_pzc_. For the composites containing diatomite in their composition, a shift in pH_pzc_ towards higher values can be observed. In addition to SiO_2_ with a strongly acidic nature [102], they can contain many mineral compounds, such as Al_2_O_3_, Fe_2_O_3_/FeO, CaO, MgO, Na_2_O, K_2_O, SO_3_, Mn_2_O_3_, P_2_O_5_, and TiO_2_. Some of them can strengthen the base properties of the composite surfaces. The mineral composition of diatomite depends on its origin and the processes it has undergone. Crystalline diatomite (CD), obtained through the calcination of diatomaceous earth, is characterized by a higher percentage of mineral compounds due to the removal of organic matter during the calcination process. All this together contributes to the relatively high pH_pzc_ value that can be observed for the ChCD composite (with crystalline diatomite), although it has the lowest percentage of chitosan component in its composition.

### 2.2. Adsorption Properties of Composites

#### 2.2.1. Adsorption Equilibrium

**Figure 8:**
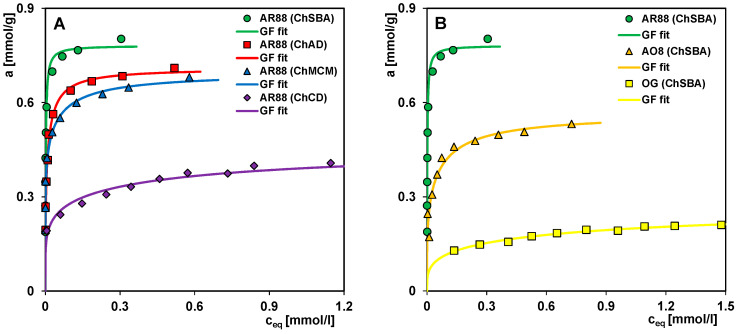
Adsorption isotherms of AR88 on the chitosan–silica composites ChSBA, ChAD, ChMCM, and ChCD (**A**). Adsorption isotherms of AR88, AO8, and OG on the composite ChSBA (**B**).

Figure 8A shows the adsorption isotherms of acid red 88 (AR88) on the chitosan–silica composites. Comparing their course and taking into account the results of the elemental analysis of composites, a correlation between the adsorption efficiency and the content of elemental nitrogen as a derivative of the nitrogen functional groups—amine and amide—originating from the biopolymer component can be observed. This confirms the hypothesis that, due to the polycationic structure of chitosan, its content in a composite largely determines the adsorption efficiency of anionic colorants. The analysis of the equilibrium data was based on the Generalized Langmuir equation (GL), which, depending on the values of the heterogeneity parameters m and n, takes simplified forms, i.e., the Langmuir equation (L) (m = n = 1), the Langmuir–Freundlich equation (LF) (0 < m = n ≤ 1), the Generalized Freundlich equation (GF) (n = 1, 0 < m ≤ 1), and the Tóth equation (T) (m = 1, 0 < n ≤ 1). The best quality of optimizations was obtained using the Generalized Freundlich (GF) equation. The values of adsorption capacity, a_m_, and equilibrium constant, log K, were calculated based on GF equation equal to 0.78 mmol/g and 2.60; 0.71 mmol/g and 1.50; 0.69 mmol/g and 0.76; and 0.46 mmol/g and −0.22 for ChSBA, ChAD, ChMCM and ChCD, respectively (Table 3). The lack of adsorption properties of silica towards acid red 88 is demonstrated in Figure S3 and the previous work by the authors [103].

Analyzing AR88 adsorption uptake, one can state that it is the highest for the three composites with the relatively high nitrogen content (1–1.3%); however, for the ChCD composite (nitrogen content 0.6%), AR88 adsorption is the lowest. Nevertheless, there is also an influence of additional features of the tested adsorbents that determine their adsorption capacity. This is evidenced by the different a_m_ values for the ChSBA and ChAD composites, which are characterized by the same nitrogen content (chitosan). To identify these features, the texture, morphology, and acid–base properties of the composites were analyzed. It appears that a more extensive pore structure of a composite provides a larger contact area of the adsorbate with the adsorption centers on the adsorbent surface and favors adsorption. While this statement is correct in the case of ChSBA, in which specific surface area and total pore area are 303 m^2^/g and 0.49 cm^3^/g, respectively, it is not entirely true for ChMCM with S_BET_ = 330 m^2^/g and V_t_ = 0.26 cm^3^/g. The parameter that limits the adsorption on ChMCM is a lower mass ratio of the organic component to the inorganic component compared to that of ChSBA, but the differentiation in pore size is the main important factor for both materials (twice the pore diameter for ChSBA facilitates and increases adsorption). In the case of ChAD, on the other hand, the above statement does not apply, as it has a structure with very low porosity, S_BET_ = 5.2 m^2^/g and V_t_ = 0.02 cm^3^/g, while its adsorption capacity is even better than that of ChMCM. It might seem that the reason for this is the presence of numerous metal oxides derived from amorphous diatomite, which after forming surface groups of -M–OH with amphoteric character, could be adsorption-active centers for anionic dyes. However, this hypothesis was disproved based on adsorption studies of acid red 88 on neat amorphous diatomite. Dye adsorption on this adsorbent practically does not occur, and similarly when using neat crystalline diatomite as an adsorbent (Figure S3). Nevertheless, the presence of metal oxides in composites containing diatomite (ChAD and ChCD) was confirmed by potentiometric titration studies, which showed a shift in their pH_pzc_ to higher values compared to the other composites (6.4 and 7.6 for ChMCM and ChAD, respectively).

Considering the effect of the different properties of the adsorbents on adsorption efficiency, an issue of the surface morphology of the composites must also be raised, in particular the degree of coverage of silica with chitosan and its structural forms. Chitosan applied to a silica matrix can form a uniformly distributed thin biofilm or locally confined compact fibers formed by the agglomerated macromolecules. Consequently, the surface morphology of the adsorbent determines the availability of a dye for the adsorption active sites (amine and amide groups).

A composite with a thin biofilm layer, where the contact surface between the adsorbate and the chitosan component is larger, can be expected to be more efficient. The question of the influence of morphological characteristics on the adsorption process for composites containing diatomite is slightly more complicated. According to the literature, raw diatomite is a sedimentary material resulting from the accumulation of skeletal remains of unicellular algae of different shapes and sizes [104]. The geometric heterogeneity of the material causes its particles to form a loose structure with a relatively low density. This property of diatomite provides better surface contact with the chitosan macromolecules in the solution phase during the impregnation process, and leads to the localization of the adsorption-active component (chitosan) in the interparticle spaces of diatomite.

The above considerations lead to the conclusion that the effectiveness of chitosan–silica composites in the adsorption of anionic dyes depends primarily on the relative contribution of chitosan in the adsorbent, but also on its porosity, the structure formed by particles (voids), the morphology of the solid surface, the particle size and geometry, and the type of biofilm coverage.

In Figure 8B, the adsorption isotherms for three sulfonated azo dyes (i.e., acid red 88 (AR88), acid orange 8 (AO8), and orange G (OG)) on the ChSBA composite are presented. The molecules of the selected adsorbates have a different chemical structure in terms of the type and number of substituents, which determines their size and physicochemical properties. The adsorption efficiency of the composite towards the pollutants decreases in the following order, AR88 > AO8 > OG, for which the parameters a_m_ and log K are equal to 0.78 mmol/g and 2.60; 0.57 mmol/g and 0.69; and 0.27 mmol/g and −0.38, respectively. The significant differences in the adsorption of these dyes can be explained by analyzing (i) their molecular form under the experimental conditions based on the data in Figure S4; (ii) their hydration capacity; and (iii) their molecular volume together with the hydration shell. Acid red 88, the adsorbate with the highest affinity to the composite surface, occurs under experimental conditions in two molecular forms with different degrees of ionization, i.e., (i) with an ionized sulfonic acid group, and (ii) with ionized sulfonic acid and hydroxyl groups. The pH of the most concentrated initial solution of AR88 was 8.0, corresponding to 82% and 18% of forms (i) and (ii), respectively. The presence of a molecular form with a higher electrical charge enhances the interactions between adsorbate and adsorbent according to the mechanism of attractive electrostatic forces and promotes adsorption. The poorer adsorption of acid orange 8 results from the lower charge of the molecule at pH ~ 6.2, which is related to the ionization of only one substituent: the sulfone group. The charge of orange G occurring under the experimental conditions (pH ~ 5.9) in the form of two ionized sulfone groups again does not appear to be the main factor determining the adsorption effectiveness. Although the molecular volume of OG is not significantly larger than the volume occupied by the molecules of other dyes (306, 305, and 281 Å^3^ for OG, AR88, and AO8, respectively), its polar surface area (PSA) is much larger (176, 111, and 111 Å^2^ for OG, AR88, and AO8, respectively) and the solubility in water (5.0, 1.5, and 1.0 g/L for OG, AR88, and AO8, respectively) indicates its high hydrophilic properties. Heteroatoms (oxygen, nitrogen, sulfur) and polar hydrogen atoms in the dye molecule can be surrounded by water clusters, which weaken the electrostatic adsorbate–adsorbent interactions and increase the actual molecular volume (together with the hydration shell). Ultimately, all this leads to a significant reduction in OG adsorption compared to other dyes.

The adsorption mechanism of all dyes on the chitosan–silica composites is mainly based on electrostatic interactions between the positively charged surface of the adsorbents and the negative ions of the adsorbates. The proportion of this type of interaction depends on the experimental conditions (pH of the initial solutions resulting from the type and amount of dye dissolved), the acid–base properties of the composites, and the acid dissociation ability of the substituents in the adsorbate molecules. The second possible type of interaction between the components of the adsorption system is hydrogen bonds resulting from the presence of amine, amide, hydroxyl, and silanol groups on the surface of the composites and azo, sulfone, methyl, and hydroxyl groups in the dye molecules. The values of the adsorption capacity and the equilibrium constant log K for the tested adsorption systems indicate a positive correlation. An increase in the adsorbent affinity to adsorbate (log K, determined by the slope of the adsorption isotherm in the range of low equilibrium concentrations) leads to the better efficiency of dye adsorption on the chitosan–silica composites. Table 4 summarizes the adsorption capacity of the most efficient materials proposed in this paper for AR88 and various other adsorbents available in the literature. One can state that the chitosan-SBA-15 composite (ChSBA) stands out from the others with its great adsorption properties, and it is inferior only to zeolite–chitosan hydrogel in adsorption performance. However, chitosan–amorphous diatomite (ChAD) and chitosan-MCM-41 (ChMCM) composites also perform quite well compared to other adsorbents.

#### 2.2.2. Adsorption Kinetics

**Figure 9:**
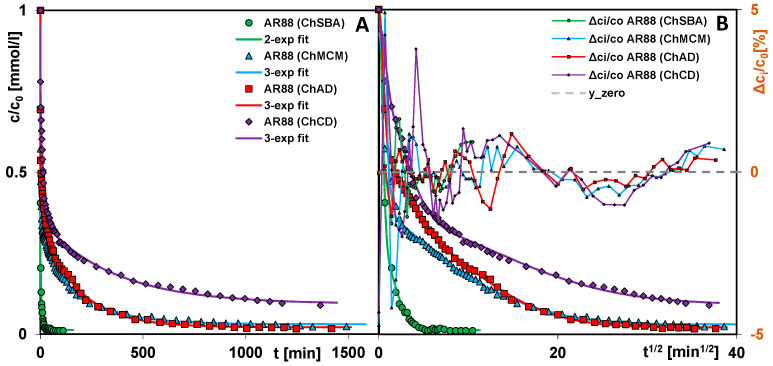
Profiles of concentration changes as a function of time (**A**) and time root (**B**) for the adsorption of AR88 on chitosan–silica composites ChSBA, ChAD, ChMCM, and ChCD.

**Figure 10:**
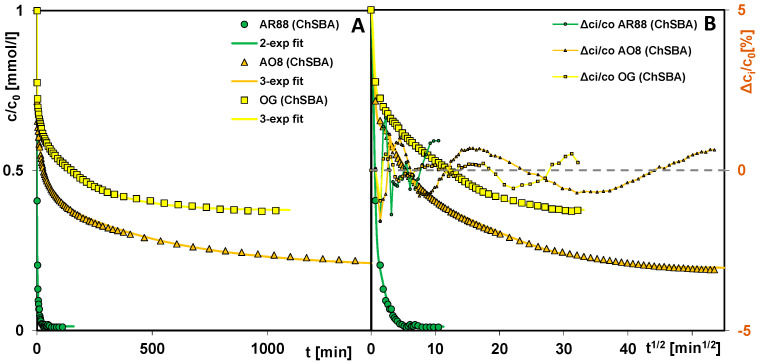
Profiles of concentration changes as a function of time (**A**) and time squared (**B**) for the adsorption of AR88, AO8, and OG on the ChSBA composite.

In order to investigate the kinetics of dye adsorption on the chitosan–silica composites, the measurements of adsorbate concentration changes in time were performed by applying a spectrophotometer with a flowing cell. For each adsorption system, a series of spectra were obtained (example in Figure S5), based on which a relative concentration profile was plotted (Figure 9A and Figure 10A). To better visualize the course of the adsorption process in the initial stage, kinetic curves were also plotted as a function of the square root of time (Figure 9B and Figure 10B). During the experiments, the mode of cyclic measurements in a feedback system was used (after each measurement, the dye solution was returned to the reaction vessel), which enabled concentration profiles to be obtained with a large number of experimental points evenly distributed on the abscissa axis.

Analyzing the curves, one can see that the course of adsorption depends on the adsorbate and the adsorbent used. In the case of AR88 adsorbed on used materials, its adsorption rate decreases in the following order: ChSBA > ChMCM ~ ChAD > ChCD. The values of parameters t_0.5 avg_ (half-time) and log k_avg_ for these systems obtained by the optimization method using the multi-exponential equation are equal to 0.35 min and 0.30; 1.53 min and −0.34; 2.84 min and −0.61; and 11 min and −1.20, respectively (Table 5). While the stage of reaching the relative concentration c/c_0_ = 0.5 is quite fast for three composites and somewhat slower for one, i.e., ChCD (but still linear in the coordinate system with the square root of time on the *X*-axis), the adsorption rate differs considerably in the subsequent stages. Among the tested systems, AR88 (ChSBA) and AR88 (ChCD) can be indicated as the most efficient and least efficient ones, respectively. The use of ChSBA as adsorbent compared to ChCD leads to a 160-times-faster decolorization of water to the 75% level (t_75%_: 1.09 and 175 min for ChSBA and ChCD, respectively) and 247 times faster to 90% (t_90%_: 5.08 and 1257 min). The total adsorbate uptake by the ChSBA, ChAD, and ChMCM composites at the time of reaching the equilibrium was close to 1, while it was 0.91 by the ChCD composite. Therefore, the parameter t_0.5 avg_ for the system with ChCD does not correspond to the expression c/c_0_ = 0.5 (as for others), but refers to a/a_0_ = 0.5.

The kinetic curves of the various dyes’ adsorption on the ChSBA composite show even greater differences than those for AR88 on the tested chitosan–silica composites. The AR88 dye is extracted the fastest (t_0.5 avg_ = 0.35 min, log k_avg_ = 0.30), with AO8 slower (t = 25 min for c/c_0_ = 0.5) and OG the slowest (t = 146 min for c/c_0_ = 0.5). With regard to the adsorption kinetics of the latter two dyes, it should be noted that although the changes in relative concentration over time are much smaller for OG, the equilibrium state of the adsorption process is achieved more quickly. Additionally, the relative uptake of OG from the solution ($u_{eq}=1-\frac{c_{eq}}{c_{0}}$) is much lower than that of AO8 and equal to 0.62 and 0.80, respectively. All this means that the parameters t_0.5 avg_ and log k_avg_ for the dyes discussed are comparable (6 min, −0.91 and 8 min, −1.07 for OG and AO8, respectively) despite the different shapes of the kinetic curves resulting from the molecular size of these dyes and from their affinity to the composite surface.

**Figure 11:**
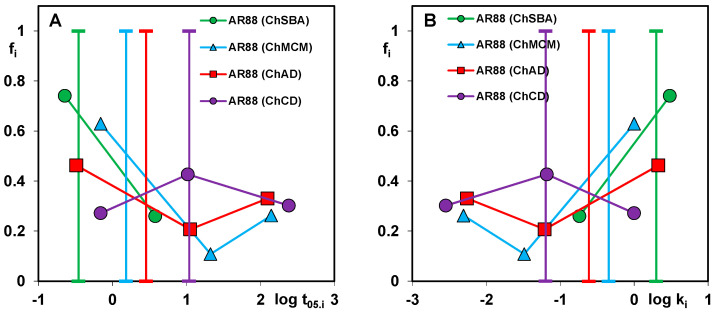

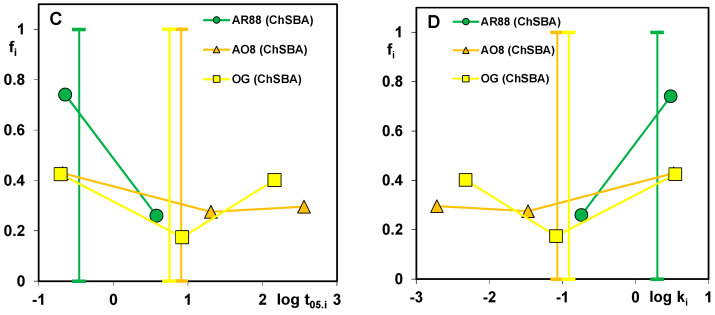
Distribution of half-time t_0.5,i_ (**A**,**C**) and rate coefficient k_i_ (**B**,**D**) for dyes’ adsorption on the chitosan–silica composites.

To optimize the kinetic data, a multi-exponential equation (m-exp) was applied which describes systems with parallel, independent processes or systems characterized by a high adsorption rate in the initial stage, followed by slower processes. Typically, such an adsorption process results from the different availability of the adsorbate for pores of different diameters and occurs when the adsorbent is a solid with a heterogeneous pore structure or a complex pore network. Since the kinetic equation discussed assumes a certain distribution of rate coefficients and an unlimited range of k_i_, its use usually gives satisfactory fitting results for many experimental systems. In the analysis of the kinetic data of dye adsorption on the chitosan–silica composites, an optimization procedure was performed using the m-exp equation with one (1-exp), two (2-exp), or three (3-exp) exponents. The most optimal variant was then selected based on the relative standard deviations SD c/c_0_. Most experimental systems were described by the equation with three exponents, while one system was described by the equation with two exponents. Each term of the equation had a specific value of k_i_, based on which the half-life t_0.5_,_i_ was calculated. The total half-life, t_0.5 avg_, was in turn calculated numerically. The adsorption kinetics for each system can be represented as a distribution of the adsorption half-times and the rate coefficients, showing the relative contribution of the slow and fast kinetic terms in the m-exp. equation (Figure 11A–D). A wider distribution of parameters means a greater variation in the rate of subsequent process stages (like during AO8 adsorption on ChSBA). A larger contribution of shorter half-times and higher rate coefficients in turn means faster kinetics (like during AR88 adsorption on ChSBA). A V-shaped distribution corresponds to kinetics with a relatively high contribution of fast and slow processes (like during the adsorption of AR88 on ChAD and OG on ChSBA).

In summary, the adsorption rate in a dye–chitosan–silica composite system depends on: (i) adsorbent composition (a higher chitosan content provides more adsorption centers and a dye can reach them more easily); (ii) the diameter and shape of adsorbent pores upon which the diffusion rate of a dye to the solid internal surface is dependent; (iii) the structure type formed by solid particles (looser structure creates voids serving as adsorption sites; (iv) the degree and morphology of chitosan coverage on a silica surface; (v) the affinity between adsorbate and adsorbent; and (vi) the dye molecule size with a solvation shell.

## 3. Materials and Methods

### 3.1. Materials

The non-ionic triblock copolymer Pluronic 123, the cationic surfactant octadecyltrimethylammonium bromide, and tetraethyl orthosilicate (TEOS ≥ 99.0%) were purchased from Sigma-Aldrich (Poznan, Poland). Ammonia water (25% pure), hydrochloric acid (35–38% pure), and acetic acid (99% pure) were purchased from Polish Chemical Reagents (POCh, Poznan, Poland). Amorphous diatomite (diatomaceous earth, natural, non-calcined, intended for consumption purposes) was supplied by FSF NatVita (Mirków, Poland). The composition of amorphous diatomite provided by the manufacturer was as follows: 89–95% amorphous silica SiO_2_, less than 1% crystalline silicon, 4% Al_2_O_3_. Crystalline diatomite (crystalline diatomaceous earth, calcined) with composition of 50–70% SiO_2_ (of which 1–10% was quartz) was purchased from Sigma-Aldrich. Chitosan derived from shrimp shells with a high quality level (QL = 200), with a degree of deacetylation of ~75% and a molecular weight of 190,000 to 370,000 Da, was purchased from Sigma–Aldrich (Poznan, Poland). Anionic dyes, i.e., acid red 88 (75% pure), acid orange 8 (65% pure), and orange G (80% pure), were purchased from Sigma-Aldrich (Poznan, Poland).

### 3.2. Synthesis of Composites

#### 3.2.1. Synthesis of SBA-15 Type Silica

Briefly, 12 g of triblock copolymer Pluronic 123 was dissolved in 360 mL of 2 M hydrochloric acid solution. Then, 90 mL of distilled water was added to the polymer solution. The flask containing the reaction mixture was transferred to a water bath (35 °C) with a mechanical stirrer, where 27 mL of TEOS was gradually added. The reaction solution was stirred at the initial temperature until a clear, homogeneous solution was obtained (for 24 h). After this time, the solution was transferred to a Teflon-lined autoclave and heated under static conditions at elevated temperatures (95 °C) for 48 h, which allowed the self-assembly of the silica nanoparticles into the phase of the desired mesoporous structure. After the hydrothermal treatment, the autoclave was cooled to room temperature and the mixture was filtered through a Buchner funnel and the resulting precipitate was washed with distilled water. In the final stage, the material was calcined in a muffle furnace (550 °C) for 5 h.

#### 3.2.2. Synthesis of MCM-41 Type Silica

Here, 8 g of surfactant (octadecyltrimethylammonium bromide) was dissolved in 360 mL of water. The flask containing the solution was transferred to a water bath heated to 35 °C with a mechanical stirrer and then 30 mL of 25% ammonia was added. After 30 min, 29.9 g of TEOS was gradually added to the reaction mixture. When the mixture became homogeneous, it was filtered through a Buchner funnel and the resulting precipitate was washed with distilled water. The material was dried in a dryer (80 °C) for 24 h and calcined in a muffle furnace (550 °C) for 5 h.

#### 3.2.3. Synthesis of Composites Based on Chitosan and Silica

Synthesized (SBA-15 and MCM-41) and commercial silica materials (amorphous diatomite and crystalline diatomite) were used as the main components of the composites. The synthesis of the composites was carried out as follows: 10 g of silica dried at 150 °C was placed in a flask containing 100 mL of a 2% acetic acid solution containing 2 g of dissolved chitosan. The flask was placed in a water bath at 40 °C and stirred with a mechanical stirrer. The solution was left under the given conditions for 24 h. After the specified time, the flask and the mixture were placed in a dryer at 60 °C and dried until the water was completely evaporated. The precipitate obtained was washed with distilled water, dried again, and ground. Depending on the type of silica component used (SBA-15, MCM-41, amorphous diatomite, crystalline diatomite), four chitosan–silica composites were obtained, which were labeled as follows in further studies: ChSBA, ChMCM, ChAD, and ChCD.

### 3.3. Adsorbate Characteristics

Three anionic dyes were used in the adsorption studies, namely acid red 88 (AR88), acid orange 8 (AO8), and orange G (OG). These substances represent the group of sulfonated azo dyes and are characterized by the physicochemical properties summarized in Table 6. The species distribution of the dyes under experimental conditions is shown in Figure S4.

### 3.4. Characterization Techniques and Calculations

#### 3.4.1. X-ray Powder Diffraction (XRPD) and Small Angle X-ray Scattering (SAXS) Analysis

X-ray powder diffraction (XRPD) analysis was performed using an Empyrean diffractometer (PANalytical, 2012, Malvern, UK) in reflection–transmission mode. X-ray tubes equipped with Cu anodes were utilized as radiation sources (X-ray wavelength (1.5418 Å)). The PIXcel3D detector was used in linear (1D) scanning mode. The incident beam path consisted of W/Si, and a stepped X-ray mirror with an elliptical shape. Scans were made over a 2θ range of 10–90° with a total exposure time of 2 h. The SAXS measurements were performed by the same device at the SAXS/WAXS sample stage with capillary mode. The device was operated with a generator setting of 40 kV and 40 mA. The incident beam path consisted of W/Si, with a stepped X-ray mirror with an elliptical shape. The SAXS measurements were performed at −0.1–4 degrees 2θ with a step size of 0.005. The primary beam was measured with a Cu 0.2 mm beam attenuator and a PIXcel3D detector. The length of the scattering vector (or scattering vector) q is defined as q = (4πsinθ)/λ, where 2θ is the scattering angle and λ is the X-ray wavelength (1.5418 Å). Background scattering was evaluated by an air scattering measurement using an empty sample holder with foil. The Dv(R) calculations were performed using the indirect Fourier transform technique applied in the EasySAXS software (PANalytical, Malvern, United Kingdom, version 1.2).

#### 3.4.2. Imaging the Morphology

The scanning electron microscopy (SEM) analysis was performed with a QuantaTM 3DFEG (FEI Company, Hillsboro, OR, USA) device working at 5 kV and a high-vacuum (8 × 10^−4^ Pa) atmosphere. Before the SEM measurements, the samples were coated with gold to improve their electrical conductivity and their ability to reflect electrons and thus provide clear images. The SEM images were colorized using MountainsLab software (v. 10, Digital Surf, Besançon, France). The Transmission Electron microscopy analysis was performed using Tecnai G2 T20 X-TWINworking at a tension of 200 kV (FEI Company, Hillsboro, OR, USA). Atomic force microscopy (AFM) analysis was performed for the illustration of 3D topography on a Bruker-Veeco-Digital Instruments Multi-Mode Atomic Force Microscope (Bruker, Bremen, Germany). The dynamic mode (tapping) was applied during AFM imaging. NanoScope Analysis software v1.40r1 from Bruker (Bruker, Bremen, Germany) was applied for the data treatment.

#### 3.4.3. Nitrogen Adsorption/Desorption Measurement

The textural evaluation of the chitosan–silica composites was obtained from the low-temperature adsorption/desorption of nitrogen using an ASAP2020 instrument (Micromeritics, Norcross, CA, USA). Based on the adsorption data, values of structural parameters, i.e., the BET specific surface area, S_BET_; the micropore surface area, S_mic_; the total pore and micropore volumes, V_t_ and V_mic_; the mean hydraulic pore diameter, D_h_; and the pore diameters from the PSD maximum from adsorption and desorption data, D_BJH ads_ and D_BJH des_, were determined. The Barrett, Joyner, and Halenda (BJH) procedure was used to calculate the pore size distributions (PSDs).

#### 3.4.4. Potentiometric Titration

Potentiometric titration was carried out in a measurement system consisting of a thermostatic vessel for sample suspension (0.1 mol/L NaCl solution as a diluent), an automatic burette (Dosimat 765, Metrohm, Herisau, Switzerland), and a pH-meter (PHM240, Radiometer, Copenhagen, Denmark). Here, 0.5 mol/L HCl and 0.5 mol/L NaOH solutions were used as an acidifying agent and a titrant, respectively. The acid–base properties of the composite surface (the point of zero charges and the surface charge densities) were determined based on the obtained data.

#### 3.4.5. Elemental Analysis

The analysis of basic elements (carbon, hydrogen, and nitrogen) in the chitosan–silica composites was performed using the Series II CHNS/O analyzer 2400 (Perkin Elmer, Waltham, MA, USA). Each sample was subjected to a combustion process at 950 °C preceded by a reduction process at 650 °C.

#### 3.4.6. Adsorption Equilibrium and Kinetics

The adsorption process for dyes on composite materials was carried out using a static method. In detail, to Erlenmeyer flasks with a series of weighed samples of the dried composite (~0.05 g), acid red 88 (AR 88) solutions of fixed concentrations (0.39–1.96 mmol/L) were added. The prepared adsorption systems were placed in a thermostatic shaker (Innova 40R model, New Brunswick, NJ, USA) and left there for 48 h under constant temperature conditions (25 °C) and stirring rate (120 rpm). Similarly, adsorption systems with acid orange 8 (AO8) and orange G (OG) were prepared, the initial concentration ranges of which were 0.36–1.80 mmol/L and 0.40–1.98, respectively. The actual dye content in the commercial product (Table 5) was taken into account in the calculations of the concentrations of the initial solutions. The pH values of the most concentrated initial solutions defined using the CPC-501 pH-meter were 8.0, 6.2, and 5.9 for AR88, AO8, and OG, respectively. The difference in adsorbate concentrations in solutions before and after the adsorption process was defined based on spectrophotometric measurements of the calibration solutions and equilibrium solutions (UV–Vis spectrophotometer Cary 4000, Varian Inc., Melbourne, VIC, Australia) at λ = 490, 505, and 475 for AO8, AR88, and OG, respectively. All equilibrium solutions were filtered before measurements. The mass balance equation was used to calculate the amount of dye adsorbed on the composite at equilibrium state, expressed in mmol/g. To analyze the adsorption data for dye-composite systems, the Generalized Langmuir (GL) equation was applied [103,118,119]:

(1) $$\theta ={\left[\frac{{\left({\mathrm{Kc}}_{\mathrm{eq}}\right)}^{n}}{1+{\left({\mathrm{Kc}}_{\mathrm{eq}}\right)}^{n}}\right]}^{m/n}$$

where θ is the global adsorption isotherm, c_eq_ is the equilibrium dye concentration (mmol/L), m and n are the heterogeneity parameters, and K is the adsorption equilibrium constant.

The Generalized Langmuir (GL) equation makes it possible to assume the specific values of m and n parameters (0 < m,n ≤ 1), which leads to four simpler equations: Langmuir (L) (GL: m = n = 1), Langmuir–Freundlich (LF) (GL: 0 < m = n ≤ 1), Generalized Freundlich (GF) (GL: n = 1, 0 < m ≤ 1), and Tóth (T) (GL: m = 1, 0 < n ≤ 1).

The kinetic measurements for dye-composite systems were performed using the technique of continuous recording of absorption spectra of the adsorbate solution. In detail: to a glass vessel with a composite sample of mass 0.05 g, 100 mL of dye solution with a concentration of 0.076 mmol/L was added. During the experiment, the reaction solution was thermostated (25 °C) (Thermostat Ecoline RE 207, Lauda, Germany) and stirred mechanically (110 rpm). At definite time intervals, absorption spectra of the dye solution (after filtration) were measured using a UV–Vis spectrophotometer (Cary 100, Varian, Melbourne, Victoria, Australia) and then returned back automatically to a glass vessel. The pH values of the initial solutions defined using the CPC-501 pH-meter were 7.6, 7.2, and 7.1 for AR88, AO8, and OG, respectively. Based on the recorded data, the concentration–time profiles were determined. To analyze the kinetic data for dye-composite systems, the multi-exponential equation (m-exp) was applied [103,120,121]:

(2) $$c=\left(c_{o}-c_{\mathrm{eq}}\right)\sum_{i=1}^{n}f_{i}\exp\left(-k_{i}t\right)+c_{\mathrm{eq}}$$

t_0.5,i_~(ln 2)/k_i_ (3)

where “i” is the term of m-exp equation, k_i_ is the rate coefficient, and u_eq_ = 1 − c_eq_/c_0_ is the relative loss of adsorbate from the solution.

## 4. Conclusions

- In this work, chitosan–silica composites were obtained by solution impregnation. Silica in the form of SBA-15, MCM-41, amorphous diatomaceous earth, and crystalline diatomaceous earth were used as the main components, which served as a matrix for the covering biopolymer and enhanced the mechanical and thermal stability of the final products. Due to the polycationic nature of chitosan, the task of this component was to improve the adsorption effectiveness towards anionic dye.
- The main factor determining the effectiveness of the adsorption of acid red 88 is the amount of biopolymer component in the composite, which provides adsorption-active centers in the form of amine and amide groups. The values of the adsorption capacity, a_m_, are as follows: 0.78 mmol/g; 0.71 mmol/g; 0.69 mmol/g for ChSBA, ChAD, and ChMCM composites (a nitrogen content between 1 and 1.3%); and 0.46 mmol/g for the ChCD composite (a nitrogen content 0.6%).
- The influence of other characteristics of the composites on the adsorption efficiency was also determined, i.e., porosity (a pronounced porosity of the material with a pore diameter that ensures free penetration by the adsorbate), the presence of inter-particle structures (interparticle spaces or voids can be filled by dye molecules), particle size and geometry (where a greater geometric heterogeneity of the material forms a looser structure by providing voids), and surface morphology—the nature of the silica covered with chitosan biofilm (a thin biofilm layer gives a larger contact area between adsorbate and adsorbent).
- The differences in the adsorption capacity of the ChSBA composite towards the dyes, acid red 88 (a_m_ = 0.78 mmol/g), acid orange (a_m_ = 0.57 mmol/g), and orange G (a_m_ = 0.27 mmol/g), were related to the physicochemical properties of the adsorbate, i.e., molecular form under experimental conditions, hydration capabilities, and the molecular volume with hydration shell.
- Kinetic studies showed that the adsorption of AR88 on the ChSBA, ChMCM, and ChAD composites was rapid, with values of the half-life parameter t_0.5_ of 0.35, 1.53, and 2.84 min, respectively. When using the ChCD composite, the process was slightly slower (t_0.5_ = 11 min). While the 100% efficiency of the AR88 adsorption process on the ChSBA composite was reached within approximately 6 min, the time required to reach half of the initial concentration was much longer in the case of AO8 and OG (25 and 146 min, respectively, and the efficiency at equilibrium was 80% and 62%, respectively).

## Figures and Tables

**Table 1:** Comparison of the texture parameters for chitosan–silica composites.

Composite	S_BET_^a^ (m^2^/g)	S_mic_^b^ (m^2^/g)	V_t_^c^ (cm^3^/g)	V_mic_^d^ (t-Plot) (cm^3^/g)	D_h_^e^ (nm)	D_BJH ads_^f^ (nm)	D_BJH des_^g^ (nm)
ChMCM	330	-	0.26	-	3.2	3.6	3.3
ChSBA	303	41	0.49	0.02	6.5	6.8	5.2
ChAD	5.2	2.4	0.02	0.001	14.5	-	-
ChCD	2.1	1.4	0.01	0.001	13.6	-	-

^a^ BET surface area calculated using experimental points at a relative pressure (p/p_0_) 0.035–0.31, where p and p_0_ are denoted as the equilibrium and saturation pressure of nitrogen. ^b^ Micropore surface area. ^c^ Total pore volume calculated by amount of nitrogen adsorbed at p/p_0_ = 0.98. ^d^ Pore volume of micropores calculated by t-plot method with fitted statistical thickness in the range of 3.56 to 4.86 Å. ^e^ Hydraulic pore diameters calculated from the BET surface areas and pore volumes according to the equation D_h_ = 4 V/S. ^f^ Pore diameter estimated from PSD maximum based on adsorption data. ^g^ Pore diameter determined from desorption data.

**Table 2:** Results of the elemental composition of the obtained composite materials.

Composite	Element Contribution		
	C (%)	H (%)	N (%)
Chitosan	40.7	7.3	7.3
ChMCM	7.1	2.1	1.0
ChSBA	8.5	2.6	1.3
ChAD	8.3	2.2	1.3
ChCD	3.3	0.8	0.6

**Table 3:** The parameters of the generalized Langmuir equation for the dye (chitosan–silica composite) systems.

System	a_m_ (mmol/g)	m	n	log K	R^2^
AR88 (ChSBA)	0.78	0.67	1	2.60	0.97
AO8 (ChSBA)	0.57	0.27	1	0.69	0.91
OG (ChSBA)	0.27	0.27	1	−0.38	0.98
AR88 (ChAD)	0.71	0.34	1	1.50	0.99
AR88 (ChMCM)	0.69	0.16	1	0.76	0.97
AR88 (ChCD)	0.46	0.18	1	−0.22	0.96

**Table 4:** Comparison of the adsorption performance of various adsorbents towards the acid red 88 dye.

Adsorbent	Adsorption Capacity, a_m_ [mmol/g]	Reference
Chitosan–nanosilica composite	0.75	[103]
Magnetic ZnFe_2_O_4_ nanoparticles	0.33	[105]
Magnetic MWCN-Fe_3_C composite	0.14	[106]
ZnO/ZnMn_2_O_4_ nanocomposite	0.19	[107]
Surfactant modified bentonite	0.23	[108]
Zeolite–chitosan hydrogel	1.02	[109]
Chitosan–silica gel composite	0.48	[110]
Chitosan–nanosilica composite	0.51	[103]
Chitosan-MCF hydrogel	0.63	[111]
Chitosan-SBA-15 composite	0.78	this paper
Chitosan–amorphous diatomite composite	0.71	this paper
Chitosan-MCM-41 composite	0.69	this paper

**Table 5:** Values of kinetic parameters determined based on the multi-exponential equation and parameters determining the quality of the applied fitting procedure.

Kinetic System	i	f_i_	log k_i_	log k_avg_	t_0.5,i_ [min]	t_0.5 avg_ [min]	t_75%_/t_90%_ [min]	u_eq_	SD (c/c_o_)	1−R^2^
AR88 (ChSBA)	1	0.74	0.48	0.30	0.23	0.35	1.09/5.8	0.99	0.74%	1.0 × 10^−3^
	2	0.26	−0.74		3.8					
AR88 (ChMCM)	1	0.63	0	−0.34	0.69	1.53	39/240	0.97	0.74%	3.9 × 10^−3^
	2	0.11	−1.49		21					
	3	0.26	−2.31		140					
AR88 (ChAD)	1	0.46	0.33	−0.61	0.33	2.84	65/255	0.98	0.50%	4.9 × 10^−4^
	2	0.21	−1.21		11					
	3	0.33	−2.26		126					
AR88 (ChCD)	1	0.27	0	−1.20	0.69	11	175/1257	0.91	0.99%	3.1 × 10^−3^
	2	0.43	−1.18		10					
	3	0.30	−2.55		244					
AO8 (ChSBA)	1	0.42	0.52	−1.07	0.21	8	770/-	0.80	0.59%	1.4 × 10^−3^
	2	0.28	−1.47		20					
	3	0.30	−2.72		362					
OG (ChSBA)	1	0.42	0.55	−0.91	0.20	6	-/-	0.62	0.31%	5.1 × 10^−4^
	2	0.18	−1.08		8					
	3	0.40	−2.32		146					

**Table 6:** Physicochemical properties of the adsorbates.

Dye Code	Chemical Formula	Dye Content [%]	Molecular Weight [g/mol]	Ionization Constant, pKa	Water Solubility [g/L]	Polar Surface Area [Å^2^]	Van Der Waals Volume [Å^3^]	Ref.
AR88	C_20_H_13_N_2_NaO_4_S	75	400.38	11.06	1.5	111	305	[112,113,114,115]
AO8	C_17_H_13_N_2_NaO_4_S	65	364.35	−1; 13.5	1.0	111	281	[112,113,115,116]
OG	C_16_H_10_N_2_Na_2_O_7_S_2_	80	452.37	12.8	5.0,7.1	176	306	[113,115,117]

## Data Availability

The data presented in this study are available on request from the corresponding author.

## Supplementary Materials

The following supporting information can be downloaded at: https://www.mdpi.com/article/10.3390/molecules29092087/s1. Figure S1: SAXS pattern of chitosan-silica composites ChSBA, ChMCM and initial SBA-15 and MCM-41 components. Figure S2: SEM images of chitosan-silica composites: ChMCM (A,B), ChSBA (C,D), ChCD (E,F) and ChAD (G,H). Figure S3: UV-Vis absorption spectra for AR88 adsorption on mesoporous silica SBA-15 (SBA) (A), mesoporous silica MCM-41 (MCM) (B), amorphous diatomite (AD) (C) and crystalline diatomite (CD) (D). (Adsorbent: m = 0.05 g; adsorbate solution: c = 0.076 mmol/L; V = 100 mL; time intervals of measurements: 10 × 1 min, 10 × 60 min). Figure S4: Percentage of the molecular forms of adsorbates under experimental pH for acid red 88 (A), acid orange 8(B), and orange G (C). Figure S5. UV-Vis absorption spectra for the exemplary adsorption system AR88 (ChAD). (Adsorbent: m = 0.05 g; adsorbate solution: c = 0.076 mmol/L; V = 100 mL; time intervals of measurements: 10 × 1 min; 10 × 3 min; 5 × 5 min; 5 × 10 min; 5 × 20 min; 18 × 60 min).
