# Supplementary material for: Synthesis of Composite Sorbents with Chitosan and Varied Silica Phases for the Adsorption of Anionic Dyes
Source: Molecules. 2024 May 1;29(9):2087. doi: 10.3390/molecules29092087 (PMC11085257; doi:10.3390/molecules29092087)
Supplement: Supplementary file 1 [file molecules-29-02087-s001.zip › molecules-2965477-supplementary.pdf]

Article

Synthesis of composites based on chitosan and various types of silica phases and their applicability in the adsorption of anionic dyes

Magdalena Blachnio, Malgorzata Zienkiewicz-Strzalka, Anna Derylo-Marczewska *

Faculty of Chemistry, Maria Curie-Sklodowska University, M. Curie-Sklodowska Sq. 3, 20-031 Lublin, Poland; magdalena.blachnio@mail.umcs.pl (M.B.); malgorzata.zienkiewicz-strzalka@mail.umcs.pl (M.Z.-S.)

* Correspondence: anna.derylo-marczewska@mail.umcs.pl (A.D.-M.) Tel.: +48-8153-755-49

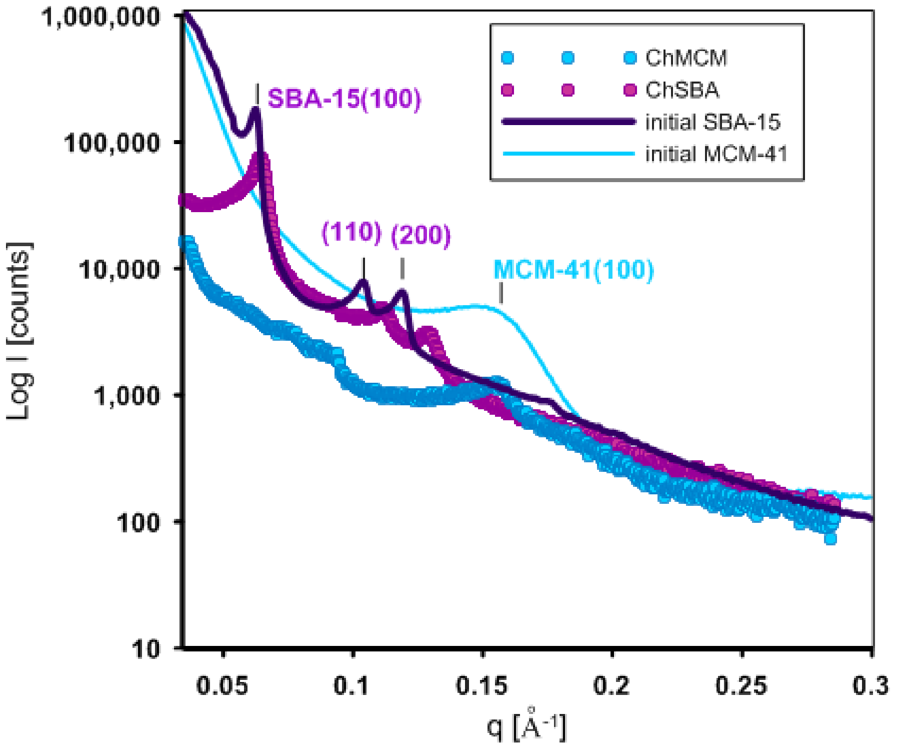

Figure S1. SAXS pattern of chitosan-silica composites ChSBA, ChMCM and initial SBA-15 and MCM-41 components.

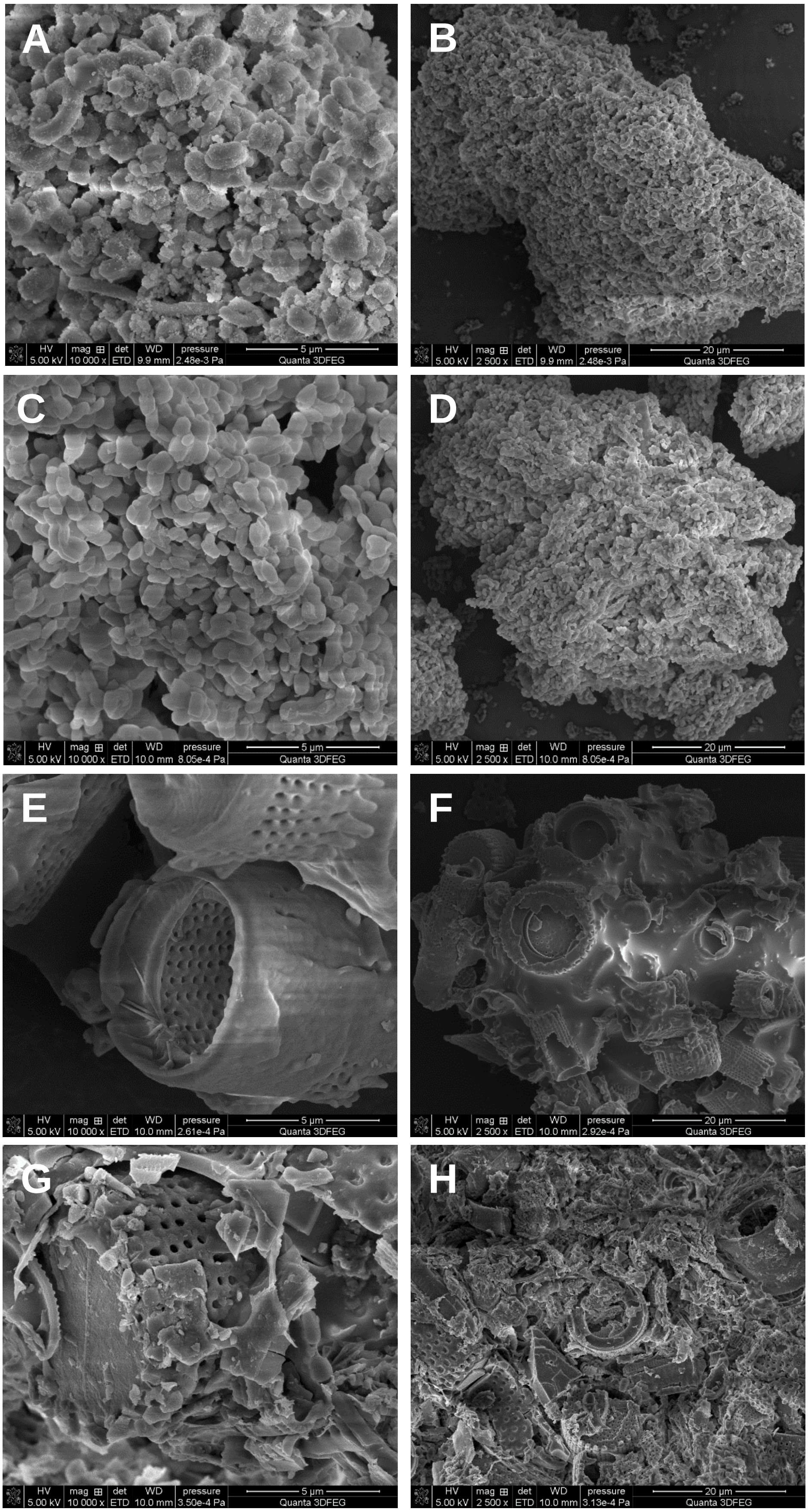

Figure S2. SEM images of chitosan-silica composites: ChMCM (**A, B**), ChSBA (**C, D**), ChCD (**E, F**) and ChAD (**G, H**).

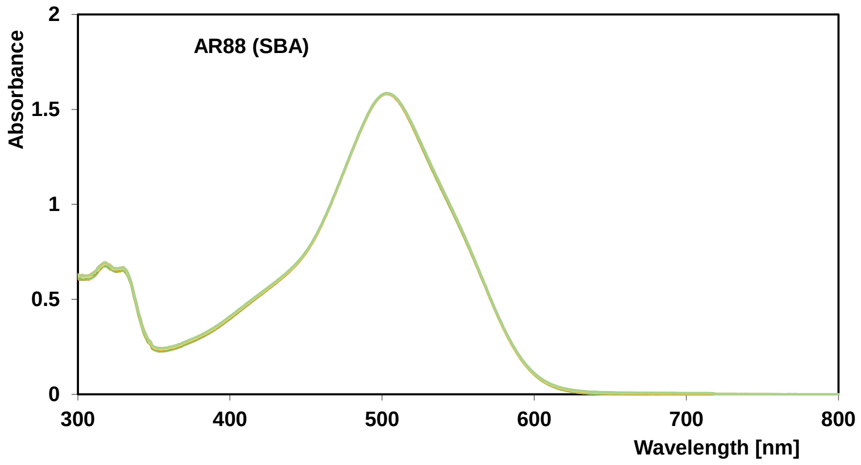
AR88 (SBA)
Absorbance
Wavelength [nm]
0
0.5
1
1.5
2
300
400
500
600
700
800

(A)

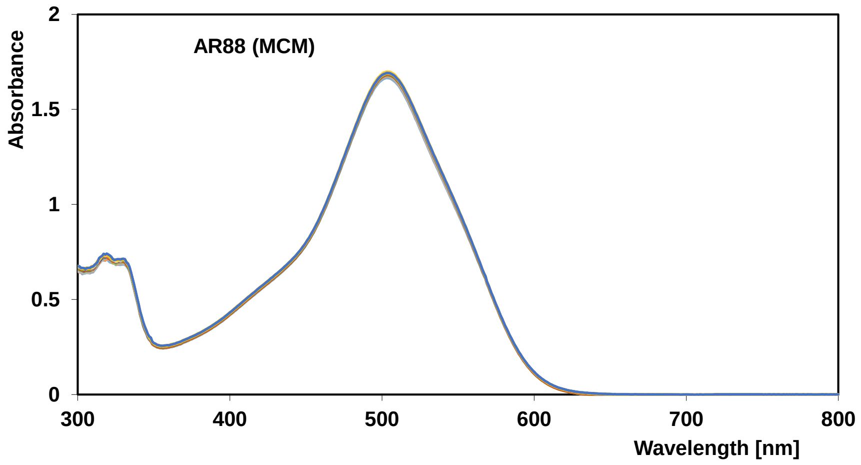
AR88 (MCM)
Absorbance
Wavelength [nm]
0
0.5
1
1.5
2
300
400
500
600
700
800

(B)

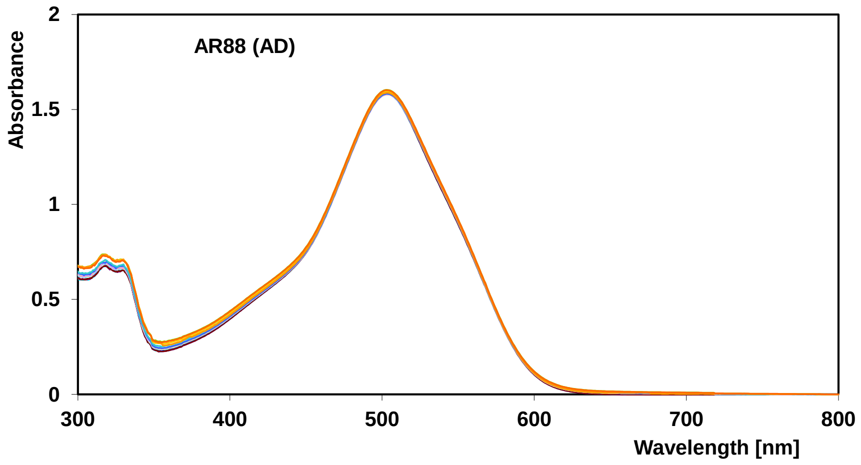
AR88 (AD)
Absorbance
Wavelength [nm]
0
0.5
1
1.5
2
300
400
500
600
700
800

(C)

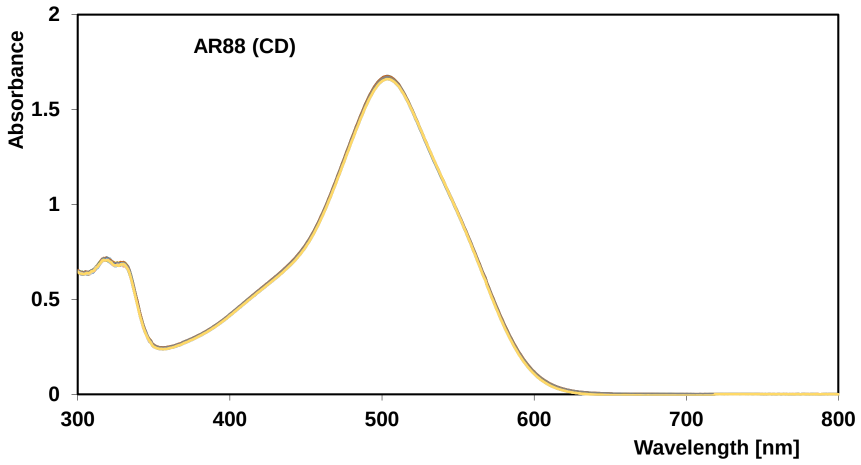

(D)

Figure S3. UV-Vis absorption spectra for AR88 adsorption on mesoporous silica SBA-15 (SBA) (**A**), mesoporous silica MCM-41 (MCM) (**B**), amorphous diatomite (AD) (**C**) and crystalline diatomite (CD) (**D**). (Adsorbent: m = 0.05 g; adsorbate solution: c = 0.076 mmol/L; V = 100 mL; time intervals of measurements: 10x1 min, 10x60 min).

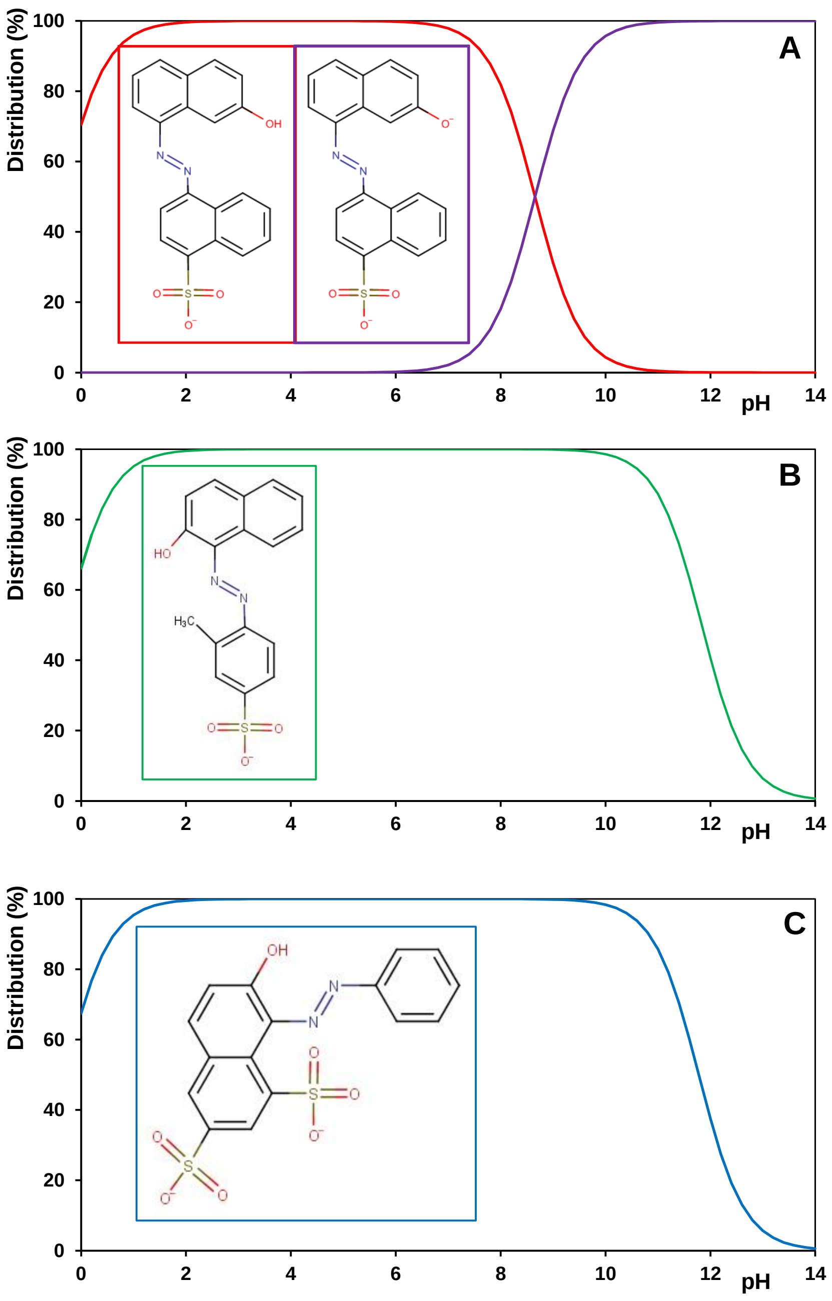

Figure S4. Percentage of the molecular forms of adsorbates under experimental pH for acid red 88 (**A**), acid orange 8(**B**), and orange G (**C**).

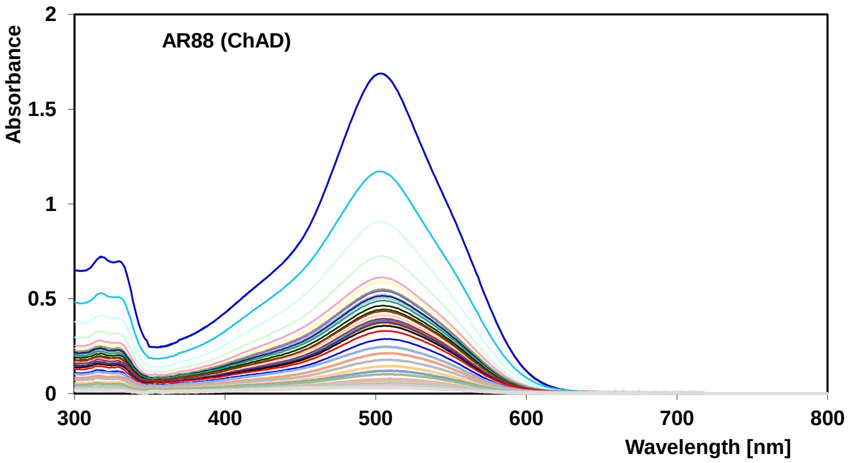

Figure S5. UV-Vis absorption spectra for the exemplary adsorption system AR88 (ChAD). (Adsorbent: m = 0.05 g; adsorbate solution: c = 0.076 mmol/L; V = 100 mL; time intervals of measurements: 10x1 min; 10x3 min; 5x5 min; 5x10 min; 5x20 min; 18x60 min).
